# A New Fractional-Order Chaotic System with Different Families of Hidden and Self-Excited Attractors

**DOI:** 10.3390/e20080564

**Published:** 2018-07-28

**Authors:** Jesus M. Munoz-Pacheco, Ernesto Zambrano-Serrano, Christos Volos, Sajad Jafari, Jacques Kengne, Karthikeyan Rajagopal

**Affiliations:** 1 Faculty of Electronics Sciences, Autonomous University of Puebla, Puebla 72000, Mexico; 2 Department of Physics, Aristotle University of Thessaloniki, 54124 Thessaloniki, Greece; 3 Department of Biomedical Engineering, Amirkabir University of Technology, Tehran 15875-4413, Iran; 4 Department of Electrical Engineering, University of Dschang, P.O. Box 134 Dschang, Cameroon; 5 Center for Nonlinear Dynamics, Defence University, P.O. Box 1041 Bishoftu, Ethiopia

**Keywords:** hidden attractor, self-excited attractor, fractional order, spectral entropy, coexistence, multistability

## Abstract

In this work, a new fractional-order chaotic system with a single parameter and four nonlinearities is introduced. One striking feature is that by varying the system parameter, the fractional-order system generates several complex dynamics: self-excited attractors, hidden attractors, and the coexistence of hidden attractors. In the family of self-excited chaotic attractors, the system has four spiral-saddle-type equilibrium points, or two nonhyperbolic equilibria. Besides, for a certain value of the parameter, a fractional-order no-equilibrium system is obtained. This no-equilibrium system presents a hidden chaotic attractor with a ‘hurricane’-like shape in the phase space. Multistability is also observed, since a hidden chaotic attractor coexists with a periodic one. The chaos generation in the new fractional-order system is demonstrated by the Lyapunov exponents method and equilibrium stability. Moreover, the complexity of the self-excited and hidden chaotic attractors is analyzed by computing their spectral entropy and Brownian-like motions. Finally, a pseudo-random number generator is designed using the hidden dynamics.

## 1. Introduction

Since Leonov et al. published their seminal paper [1], the attractors in dynamical systems have been categorized as self-excited attractors and hidden attractors. A self-excited attractor has a basin of attraction that is associated with an unstable equilibrium, the most of common examples of integer-order chaotic flows showing self-excited attractors are Lorenz, Chen, Rössler, and Lü systems, among many others [2,3,4,5]. Conversely, an attractor is called hidden if its basin of attraction does not intersect with small neighborhoods of the unstable equilibrium [6]. Additionally, the attractors in dynamical systems with no-equilibrium, with curves and surfaces of equilibria, and with stable equilibria also belong to the category of hidden attractors [1,6]. Hidden attractors are very important in engineering applications because they allow the study and understanding of the unexpected and potentially disastrous responses of the dynamical systems to perturbations, for instance, in mechanical structures, like a bridge or airplane wings [7,8,9], aircraft control systems [10], PLL circuits [1], drilling systems with induction motors [11], and secure communication schemes [1,12]. Hence, numerous integer-order chaotic flows with hidden attractors have been proposed [7,13,14,15,16,17,18,19,20,21,22,23,24].

However, it should be noted that most of the studies about hidden attractors have mainly concentrated on continuous-time dynamical systems of integer-order. In recent years, fractional calculus has received much attention due to fractional derivatives providing more accurate models than their integer-order counterparts. Many examples have been found in different interdisciplinary fields [25], ranging from the description of viscoelastic anomalous diffusion in complex liquids, D-decomposition technique for control problems, chaotic systems; to macroeconomic models with dynamic memory, forecast of the trend of complex systems, and so on [26,27,28,29,30,31,32,33,34]. Those works have demonstrated that fractional derivatives provide an excellent approach to describing the memory and hereditary properties of real physical phenomena.

Therefore, the research effort oriented to hidden attractors in fractional-order dynamical systems is vital to understand this exciting and still less-explored subject of importance. In the literature, few works have reported hidden attractors in fractional-order dynamical systems with one stable equilibrium [35,36], with no-equilibria [37,38,39,40], with a line or surfaces of equilibria [41,42], or even in fractional-order hyperchaotic systems [43,44]. However, those fractional order systems generate only one family of hidden attractors, i.e., line, surface, stable, and without equilibrium. A remaining research question is whether fractional-order dynamical systems whose dynamics can generate both self-excited and hidden attractors could exist. The first response was recently proposed by Rajagopal et al. [45] through a dynamical system and its fractional-order form, which changes from hidden to self-excited attractors and vice versa by modifying two system parameters.

Motivated by the aforementioned discussion, in this paper, we propose a new fractional-order dynamical system with four nonlinearities and a single system parameter. One salient feature of this fractional-order system is that it generates different families of self-excited and hidden attractors as a function of only one parameter. This parameter performs as a constant controller to select the required dynamics. More specifically, the proposed system exhibits a typical self-excited chaotic attractor with four equilibrium points of the type spiral saddle index 1 and index 2. Moreover, the proposed system has a self-excited chaotic attractor coexisting with two nonhyperbolic equilibrium points. A nonhyperbolic type of chaos is unusual because it does not satisfy the Shilnikov theorems.

Surprisingly, the proposed fractional-order system also has a hidden chaotic attractor without equilibria. Unlike other approaches, the resulting hidden attractor can be observed in a fractional order as low as 0.95. Finally, the multistability phenomenon was also found in the fractional-order no-equilibrium system. Multistability leads to different qualitative behavior in a given nonlinear dynamical system for the same parameter values. In the proposed system, a hidden chaotic attractor coexist with a periodic attractor. Since the system equations contain no unnecessary terms and the system parameter has a minimum of digits, the proposed fractional-order system can be considered elegant in the sense of Sprott [46]. Moreover, the criterion (iii) in [47] for reporting a new chaotic system is also satisfied. The multiple complex dynamics of the proposed system were studied by applying a numerical simulation approach to compute the Lyapunov exponents, basins of attraction, bifurcation diagrams, and phase portraits. Additionally, the 0–1 test was employed to detect a Brownian-like motion in the fractional-order system.

The complexity measure is an important property to characterize the dynamics of a chaotic system; it can also be used as the core in many applications of information security. The complexity is obtained using the spectral entropy for both self-excited and hidden attractors. From the spectral entropy analysis, the time series of the hidden attractor is used to design a pseudo-random number generator (PRNG).

The rest of this paper is organized as follows. Section 2 provides the mathematical background related to fractional calculus. Section 3 presents the new fractional-order system, along with the mechanism employed to get the hidden and self-excited attractors. Section 4 shows the results of the 0–1 test algorithm and spectral entropy. Section 5 reports the design of PRNG. Finally, Section 6 summarizes the conclusions.

## 2. Mathematical Background

In this section, we provide the background to support our main results. The integro-differential operator, denoted as ${}_{a}D_{t}^{q}$, is a combined differentiation and integration operator commonly used in fractional calculus. This operator is a notation for taking both the fractional derivative and the fractional integral of a function, combining them into a single expression that can be formally defined as

(1) $${}_{a}D_{t}^{q}f=\left\{\begin{array}{cc} \frac{d^{q}f}{dt^{q}}, & q>0,\\ f, & q=0,\\ \int_{a}^{t}f{\left(d\tau \right)}^{q}, & q<0, \end{array}\right.$$

where *f* is a function of time, *a* and *t* are the limits of the operation, and q∈R is the fractional order. As we know now, there are several different definitions for the fractional differential operator that can be adopted for (1). Hereafter, we consider the fractional derivative operator $d^{q}/dt^{q}$, with m−1<q≤m∈N, to be Caputo’s derivative [48], with starting point a=0, defined by

(2) $$D_{t}^{q}f\left(t\right)=\frac{1}{\Gamma (m-q)}\int_{0}^{t}\frac{f^{\left(m\right)}\left(\tau \right)}{{\left(t-\tau \right)}^{q+1-m}}d\tau ,$$

where *m* is an integer number and Γ(·) is the gamma function. Caputo’s derivative of order *q* is a formal generalization of the integer derivative under the Laplace transformation, and it is widely used in engineering [49].

### 2.1. Predictor–Corrector Scheme

The numerical method used in this work to compute the solution of the fractional-order system is the Adams–Bashforth–Moulton (ABM) predictor–corrector scheme, reported in [50,51,52]. The predictor–corrector scheme is based on the Caputo fractional differential operator (2), which allows us to specify both homogeneous and inhomogeneous initial conditions.

Consider the following fractional differential equation:

(3) $$\begin{array}{cc} D^{q}y\left(t\right) & =f(t,y(t)),\;0\leq t\leq T;\\ y^{\left(k\right)}\left(0\right) & =y_{0}^{\left(k\right)},\;\;k=0,1,\ldots ,n-1. \end{array}$$

The solution of (3) is given by an integral equation of Volterra type as

(4) $$y\left(t\right)=\sum_{k=0}^{\lceil q\rceil -1}y_{0}^{k}\frac{t^{k}}{k!}+\frac{1}{\Gamma (q)}\int_{0}^{t}{\left(t-z\right)}^{q-1}f\left(z,y\left(z\right)\right)dz.$$

As it is shown in [50], there is a unique solution of (3), within an interval [0,T], thence we are interested in a numerical solution on the uniform grid $\left\{t_{n}=nh|n=0,1,\ldots ,N\right\}$ with an integer *N* and stepsize h=T/N. Then, (4) can be replaced by a discrete form to get the corrector, as follows

(5) $$\begin{array}{ccc} y_{h}\left(t_{n+1}\right) & = & \sum_{k=0}^{\lceil q\rceil -1}y_{0}^{k}\frac{t^{k}}{k!}+\frac{h^{q}}{\Gamma (q+2)}f\left(t_{n+1},y_{h}^{p}\left(t_{n+1}\right)\right)\\ & & \;+\frac{h^{q}}{\Gamma (q+2)}\sum_{j=0}^{n}a_{j,n+1}f\left(t_{j},y_{h}\left(t_{j}\right)\right), \end{array}$$

where

(6) $$a_{j,n+1}=\left\{\begin{array}{c} n^{q+1}-\left(n-q\right){\left(n+1\right)}^{q},\;j=0,\\ {\left(n-j+2\right)}^{q+1}+{\left(n-j\right)}^{q+1}\\ -2{\left(n-j+1\right)}^{q+1},\;1\leq j\leq n,\\ 1,\;j=n+1, \end{array}\right.$$

Moreover, the predictor has the following structure

(7) $$y_{h}^{p}\left(t_{n+1}\right)=\sum_{k=0}^{\lceil q\rceil -1}y_{0}^{k}\frac{t^{k}}{k!}+\frac{1}{\Gamma (q)}\sum_{j=0}^{n}b_{j,n+1}f\left(t_{j},y_{h}\left(t_{j}\right)\right),$$

with $b_{j,n+1}$ defined by

(8) $$b_{j,n+1}=\frac{h^{q}}{q}\left({\left(n+1-j\right)}^{q}-\left(n-j^{q}\right)\right).$$

The error of this approximation is given by

(9) $$\max_{j=0,1,\ldots N}|y\left(t_{j}\right)-y_{h}\left(t_{j}\right))|=O\left(h^{P}\right),$$

where P=min(2,1+q).

### 2.2. Stability of Fractional-Order Systems

This subsection presents several definitions for the stability of fractional-order autonomous systems. Starting from Equations (1) and (2), it is possible to study the stability of fractional-order systems. A fractional-order differential equation with 0<q<1 typically presents a stability region that is larger than that of the same equation with integer order q=1.

**Definition** **1.** *The roots of the equation f(x)=0 are called the equilibria of the fractional-order differential system $D^{q}x=f(x)$, where $x={\left(x_{1},x_{2},\ldots ,x_{n}\right)}^{T}\in R$, f(x)∈R and $D^{q}x={\left(D^{q_{1}}x_{1},D^{q_{2}}x_{2},\ldots ,D^{q_{n}}x_{n}\right)}^{T}$, $q_{i}\in R^{+}$, i=1,2,…,n.*

**Theorem** **1.** *Consider a commensurate-order system described by*

(10) $$D^{q}x=\mathrm{Ax},\;x\left(0\right)=x_{0}$$

*with 0<q<1, $x\in R^{n}$ and $A\in R^{n\times n}$. It has been shown [53,54,55,56,57,58] that this fractional order system is asymptotically stable if and only if the following condition is satisfied*

(11) |arg(λ)|>qπ/2,

*where |arg(λ)| represents all eigenvalues of A. Besides, the critical eigenvalues of A satisfying |arg(λ)| = qπ/2 must have a geometric multiplicity of one, which stands for the dimension of subspace of v for Av=λv.*

**Theorem** **2.** *Consider an incommensurate-order system described by*

(12) $$D^{q}x=\mathrm{Ax},\;x\left(0\right)=x_{0}$$

*where $x={\left(x_{1},x_{2},\ldots ,x_{n}\right)}^{T}\in R$, $D^{q}x={\left(D^{q_{1}}x_{1},D^{q_{2}}x_{2},\ldots ,D^{q_{n}}x_{n}\right)}^{T}$, $q_{i}\in R^{+}$, i=1,2,…,n, $0<q_{i}<1$, and $A=\left(a_{ij}\right)\in R^{n\times n}$, i=1,2,…,n, j=1,2,…,n. By assuming w as the lowest common multiple of the denominators $u_{i}$ of $q_{i}$, where $q_{i}=v_{i}/u_{i}$, $(u_{i},v_{i})=1$, $u_{i},v_{i}\in Z^{+}$ for i=1,2,…,n, the characteristic matrix of (12) is defined by*

(13) $$\Delta \left(\lambda \right)=\left[\begin{array}{cccc} \lambda^{wq_{1}}-a_{11} & -a_{12} & \cdots & -a_{1n}\\ -a_{21} & \lambda^{wq_{2}}-a_{22} & \cdots & -a_{2n}\\ \vdots & \vdots & \ddots & \vdots \\ -a_{n1} & -a_{n2} & \cdots & \lambda^{wq_{n}}-a_{nn} \end{array}\right].$$

*Then, the system (12) is globally asymptotically stable in the Lyapunov sense if all roots λ of its characteristic polynomial, given by equation det(Δ(λ))=0, satisfy |arg(λ)|>π/2w [53,54,55,56,57,58].*

**Theorem** **3.** *The equilibrium point $E_{*}$ is asymptotically stable if and only if the instability measure*

(14) $$\rho =\left(\pi /2w\right)-\min_{i}\left\{\arg\left(\lambda_{i}\right)\right\}$$

*is strictly negative, where the $\lambda_{i}$ parameters are roots of equations: $\det(\mathrm{diag}\left(\left[\lambda^{wq_{1}}\;\lambda^{wq_{2}}\cdots \lambda^{wq_{n}}\right]\right)-\partial f/\partial x{|}_{x=E_{*}})=0$, $\forall E_{*}\in \Omega$ [57,58]. If ρ≥0 and the critical eigenvalues satisfying ρ=0 have the geometric multiplicity one, then $E_{*}$ is stable.*

**Remark** **1.** *If ρ is positive, then $E_{*}$ is unstable and the system may exhibit chaotic behavior [57,58].*

## 3. A New Three-Dimensional Fractional-Order Chaotic System

Recently, Munoz-Pacheco et al. [59] proposed a fractional-order dynamical system with a line, lattice, and 3D grid of boostable variables. The chaotic attractors of that system are self-excited. Inspired from that work, we propose a new fractional-order chaotic system given by

(15) $$\begin{array}{ccc} D^{q_{1}}x & = & yz+x(y-a),\\ D^{q_{2}}y & = & 1-|x|,\\ D^{q_{3}}z & = & -xy-z, \end{array}$$

where *a* is a real parameter, $\left(q_{1},q_{2},q_{3}\right)\in \left[0,1\right]$ are the fractional-order derivatives, and x,y,z are the states’ variables. In the fractional-order system (15), the Caputo definition of fractional-order derivative (2) is used. The fractional-order system (15) presents a unique characteristic. The parameter *a* behaves as a controller of the diverse complex dynamics generated by the system, such as hidden and self-excited attractors. Therefore, the fractional-order system (15) belongs to different classes of dynamical systems, i.e., a new class of systems without equilibrium, a new class of systems with multistability, a subclass of systems with nonhyperbolic equilibria, and the well-known class of systems of the hyperbolic type. To the best knowledge of the authors, this is the first time reporting a fractional-order chaotic system that presents the unique characteristic of switching from self-excited chaotic attractors to hidden chaotic attractors, and the coexistence of hidden attractors which arise by varying just one single parameter. Also, the hidden chaotic attractor can be observed with a fractional order as low as q=0.95.

In this manner, the study conducted herein could be straightforwardly expanded to find other fractional-order systems, with one single parameter generating different families of hidden and self-excited attractors, by applying a systematic computer search similar to [7,16].

### 3.1. Self-Excited Chaotic Attractor: Spiral Saddle Type of Equilibrium Points

In order to obtain the equilibrium points of the system (15), the left-hand side of the system is kept at zero, so the system’s equations can be written as

(16) $$\begin{array}{ccc} 0 & = & yz+x(y-a),\\ 0 & = & 1-|x|,\\ 0 & = & -xy-z. \end{array}$$

The equilibria $E_{*}=\left(x_{*},y_{*},z_{*}\right)$ of the system (16) are

(17) $$\begin{array}{c} E_{1}=\left(1,\left(1+\sqrt{1-4a}\right)/2,-\left(1+\sqrt{1-4a}\right)/2\right),\\ E_{2}=\left(-1,\left(1-\sqrt{1-4a}\right)/2,\left(1-\sqrt{1-4a}\right)/2\right),\\ E_{3}=\left(1,\left(1-\sqrt{1-4a}\right)/2,-\left(1-\sqrt{1-4a}\right)/2\right),\\ E_{4}=\left(-1,\left(1+\sqrt{1-4a}\right)/2,\left(1+\sqrt{1-4a}\right)/2\right). \end{array}$$

As can be seen from (17), the system parameter *a* is a controller for the kind of equilibria, i.e., the parameter *a* is also known as a bifurcation parameter. In this case, a self-excited attractor can be observed when a<1/4. Let a=−1, then the equilibrium points $E_{*}$ are as given in Table 1. For investigating the stability and type of these equilibrium points, the Jacobian matrix of system (16) is defined by

(18) $$J=\left[\begin{array}{ccc} y+1 & x+z & y\\ -sign(x) & 0 & 0\\ -y & -x & -1 \end{array}\right],$$

where the resulting eigenvalues evaluated at $E_{*}$ are as shown in Table 1. Therefore, the fractional-order system (16) has four hyperbolic equilibrium points of the type spiral saddle index 1 and index 2, where the index is the number of eigenvalues with a positive real part, respectively. According to Theorem 1, the fractional-order system is asymptotically stable if q<0.9010.

**Lemma** **1.** *When q=0.93 and a=−1, the system (15) exhibits a self-excited chaotic attractor.*

**Proof.**  In order to generate a chaotic behavior in the system (15), the instability measure ρ defined in Theorem 3 must be positive. By selecting q=0.93, a=−1, and w=100, the characteristic equation of the equilibrium points $E_{1}$ and $E_{4}$ is

(19) $$\lambda^{279}-1.6180\lambda^{186}-0.6180\lambda^{93}-2.2360,$$

with unstable root λ=1.0090, while the characteristic equation at the equilibria $E_{2}$ and $E_{3}$ is

(20) $$\lambda^{279}+0.6180\lambda^{186}+1.6180\lambda^{93}+2.2360,$$

with unstable roots $\lambda_{1,2}=1.0039\pm 0.0153i$. Then, the instability measure of the system is ρ=(π/2m)−0.0152>0. Therefore, the fractional-order system (15) satisfies the necessary condition for exhibiting a self-excited chaotic attractor when q=0.93 and a=−1. ☐

**Figure 1:**
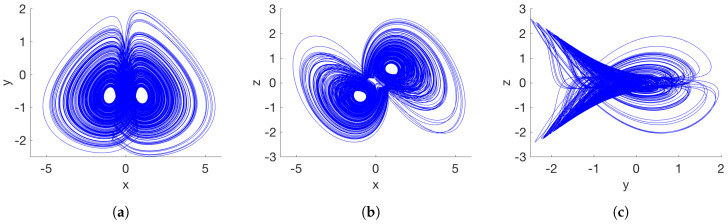
Self-excited attractor of the system (15) considering a=−1 and q=0.93. (**a**) x–y plane; (**b**) x–z plane; (**c**) y–z plane.

Numerical simulation results in Figure 1 illustrate the existence of a chaotic attractor for the given fractional order. All numerical analyses presented herein were obtained by the Adams–Bashforth–Moulton predictor–corrector scheme of Section 2.1, with h=0.01.

To verify whether the system (15) is chaotic in the classical sense, its Lyapunov exponents are calculated. The Lyapunov exponents (LEs) are indicated by $LE_{1}$, $LE_{2}$, and $LE_{3}$ in Table 1. As is well known, a system is considered chaotic if $LE_{1}>0$, $LE_{2}=0$, $LE_{3}<0$ with $|LE_{1}|<|LE_{3}|$. Time series-based LEs calculation methods, like Wolf algorithm [60], Jacobian method [61], and neural network algorithm [62], are popular known ways of calculating Lyapunov exponents for integer and fractional-order systems. The Wolf algorithm [60] is used herein to calculate the LEs.

### 3.2. Degenerate Case: Self-Excited Chaotic Attractor with Nonhyperbolic Equilibria

A nonhyperbolic equilibrium point has one or more eigenvalues with a zero real part. In three-dimensional systems, 11 combinations can be determined [63]. Among them, six have only real eigenvalues, five present eigenvalues with a complex conjugate pair and one real part, and only two do not have nonzero real eigenvalues. Therefore, the stability of systems with nonhyperbolic equilibria cannot be obtained from their eigenvalues, because there is not an eigenvalue with a positive real part. Such systems can have neither homoclinic nor heteroclinic orbits, and thus the Shilnikov method cannot be used to verify the chaos [64]. Very few examples of fractional-order systems with nonhyperbolic equilibria have been previously reported.

**Figure 2:**
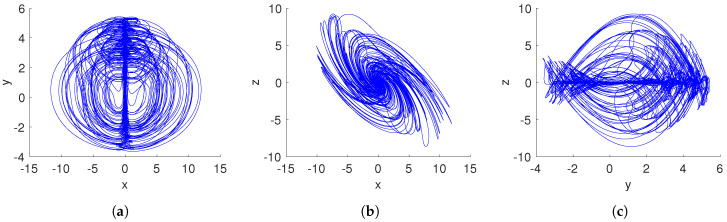
Chaotic attractor of the system (15) with nonhyperbolic equilibrium points, a=0.25 and q=0.99. (**a**) x–y plane; (**b**) x–z plane; (**c**) y–z plane.

As given in Table 1, the proposed fractional-order system (15) has two nonhyperbolic equilibrium points when parameter a=1/4. The equilibria have a zero real eigenvalue and two complex conjugate eigenvalues with a negative real part. Therefore, the resulting self-excited attractor is of a nonhyperbolic type of chaos. Figure 2 shows the phase portraits.

**Figure 3:**
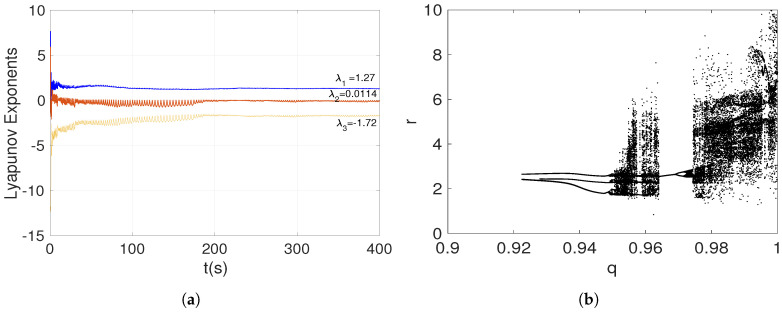
(**a**) Lyapunov exponents spectrum, and (**b**) bifurcation diagram of the fractional-order nonhyperbolic system (15) when a=1/4.

Figure 3a shows the Lyapunov exponents spectrum when the fractional-order system (15) is nonhyperbolic. The positive Lyapunov exponent indicates a chaotic behavior. Additionally, the dynamical behavior of the system (15) can also be illustrated by the bifurcation diagram in Figure 3b. Due to system (15) having only one parameter, which must be a=1/4 to present nonhyperbolic equilibrium points, it is interesting to analyze its dynamical behavior when *a* is fixed and the fractional-order *q* is varied. The bifurcation diagram in Figure 3b demonstrates a period-doubling route to chaos.

### 3.3. Hidden Chaotic Attractor Localization in the Fractional-Order System without Equilibria

Most familiar examples of low-dimensional chaotic flows occur in systems having one or more saddle points. However, further studies showed that the self-excited periodic and chaotic oscillations did not give exhaustive information about the possible types of oscillations, i.e., “hidden oscillations” and “hidden attractors”. So, this class of attractors should be introduced according to the following definition:

**Definition** **2.** *An attractor is called a self-excited attractor if its basin of attraction intersects with any open neighborhood of an equilibrium, otherwise it is called a hidden attractor [1,6].*

With equilibrium, we are stating the equilibrium points of the state variables. Definition 2 also includes fractional-order dynamical systems with no-equilibria, line and surfaces of equilibria, and stable equilibria [35,36,37,38,39,40,41,42].

**Figure 4:**
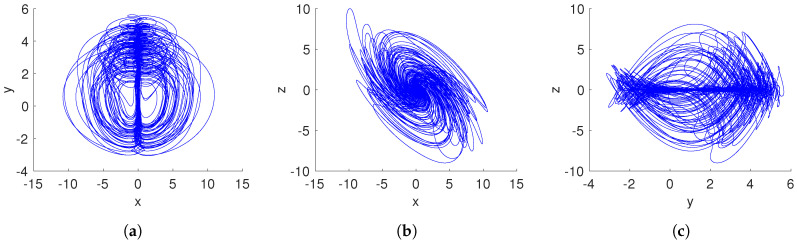
Hidden attractor of the system (15) considering a=0.35, q=0.97, and initial conditions (x(0),y(0),z(0))=(1,1,1). (**a**) x–y plane; (**b**) x–z plane; (**c**) y–z plane.

**Figure 5:**
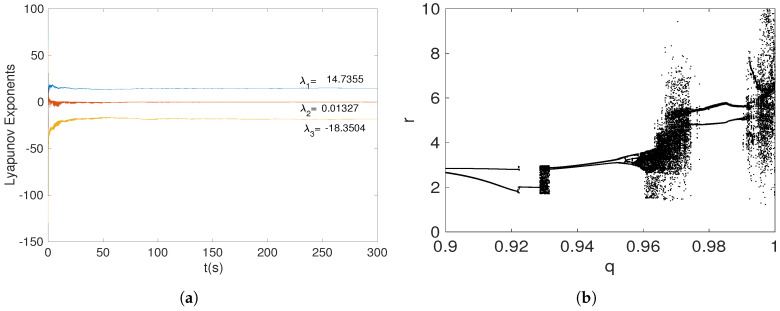
(**a**) Lyapunov exponents spectrum, and (**b**) bifurcation diagram of the fractional-order no-equilibrium system (15), when a>1/4.

Similar to aforementioned scenarios, the parameter *a* is a controller of the dynamical behavior of the proposed system (15). In this case, if a>1/4, a fractional-order system without equilibrium points is obtained. Hence, the resulting attractor is hidden using Definition 2. By selecting a=0.35, and the fractional-order q=0.97, the proposed system (15) generates the hidden chaotic attractor shown in Figure 4. It is important to note that the shape of the chaotic attractor in the x–z plane is similar to a hurricane. Moreover, the chaos generation is demonstrated by the Lyapunov exponents spectrum given in Figure 5a. As stated in Table 1, the largest Lyapunov exponent $LE_{1}$ is positive, and $|LE_{1}|<|LE_{3}|$, indicating a chaotic behavior.

By using the fractional-order *q* as bifurcation parameter, the bifurcation diagram of system (15) when it generates a hidden attractor (a>1/4) is illustrated in Figure 5b. As can be seen from the bifurcation diagram, there are three regions where the chaotic behavior emerged, i.e., for 0.9285<q<0.931, 0.962<q<0.973, and q>0.9955, a hidden attractor can be observed. This result indicates that the hidden chaotic attractor depends on the selected fractional order.

### 3.4. Coexistence of Hidden Attractors Regimes in the Fractional-Order System without Equilibria

The coexistence of attractors means that two or more different attractors are generated in a dynamical system from different initial conditions, which is an important and interesting nonlinear phenomenon [18,65]. In this subsection, we focus on studying the coexisting hidden attractors of the fractional-order no-equilibrium system (15). A necessary tool for analyzing the coexistence of attractors is the basin of attraction. All attractors, whether they be stable equilibria, limit cycles, attracting tori, or hidden strange attractors, are surrounded by a basin of attraction representing the set of initial conditions in the state space whose orbits approach and map out the attractor as time approaches infinity [66].

**Figure 6:**
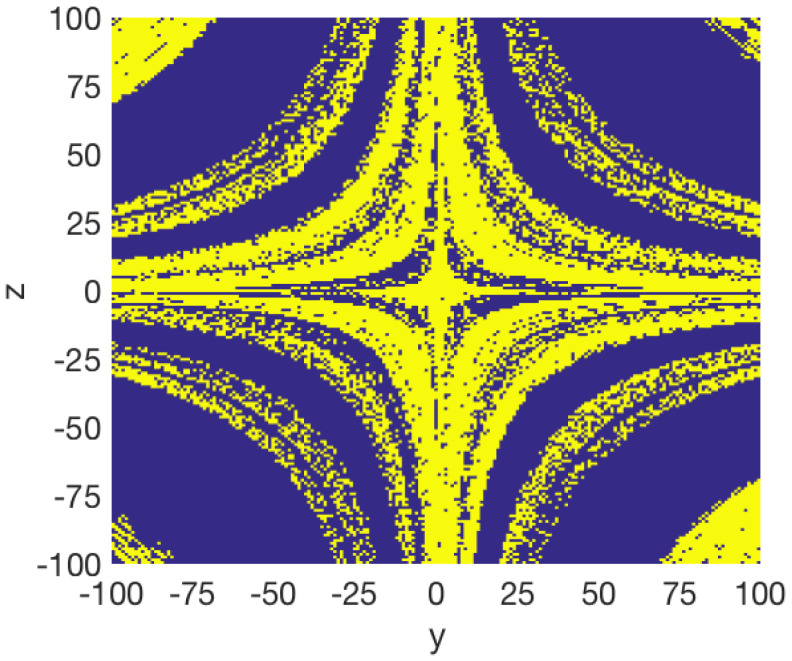
Cross-section of the basins of attraction of the two coexisting attractors in the y–z plane at x=0 for the fractional-order chaotic system without equilibrium (15) when a=0.35 and q=0.996.

**Figure 7:**
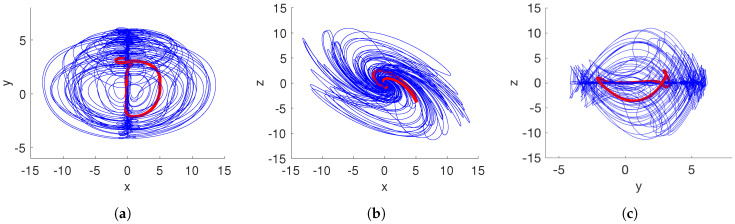
Coexistence of hidden chaotic and periodic attractors of the system (15) considering a=0.35 and q=0.996. (**a**) x–y plane; (**b**) x–z plane; (**c**) y–z plane.

**Figure 8:**
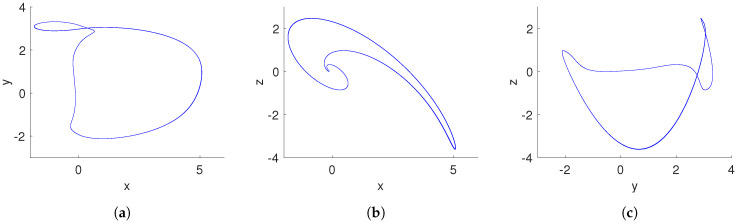
Hidden periodic attractor of the fractional-order system (15) with a=0.35, q=0.996, and initial conditions (x(0),y(0),z(0))=(0,75,−50). (**a**) x–y plane; (**b**) x–z plane; (**c**) y–z plane.

Figure 6 shows the basins of attraction of the system (15) for the cross-section in the y–z plane at x=0 with a=0.35 and q=0.996. We found that the initial conditions inside of the yellow region converge to a hidden chaotic attractor, as shown in Figure 7, whereas the initial conditions belonging to the blue region lead to a hidden periodic attractor, as shown in Figure 8. This result confirms that there are two different hidden attractors coexisting in the proposed fractional-order chaotic system (15). Both coexisting attractors are also shown in Figure 7. Besides, this behavior also indicates multistability, because different initial conditions converge to different hidden attractors.

Table 1 gives the Lyapunov exponents spectrum for both hidden chaotic and periodic attractors, respectively. The positive, zero, and negative Lyapunov exponents of the hidden chaotic attractor indicate chaotic behavior, while a zero and two negative Lyapunov exponents point out a hidden periodic attractor.

### 3.5. Mechanism of the Different Dynamics

The mechanism of generating several types of equilibria in the proposed fractional-order system (15) is simple and intuitive. The basic idea consists of varying the single system parameter *a* in a range from negative to positive values, similar to the bifurcation analysis for integer-order systems. By analyzing the symbolic equation of the equilibrium points (17), we realized that the number and stability of equilibria can be changed with the parameter *a*. One can easily see that system (15) has four unstable equilibrium points (spiral saddle index 1 and index 2) when a<1/4. As a result, the fractional-order system (15) can be defined into a class of fractional-order chaotic systems with hyperbolic equilibrium points, which is the most typical form obtained for a chaotic attractor.

Next, with a=1/4, the fractional-order system (15) degenerates, in the sense that their Jacobian eigenvalues at the equilibria consist of one zero eigenvalue and a complex conjugate pair with a negative real part. Clearly, the corresponding two equilibria are nonhyperbolic. Hence, the system (15) belongs to a subclass of fractional-order chaotic systems with nonhyperbolic equilibrium points.

**Figure 9:**
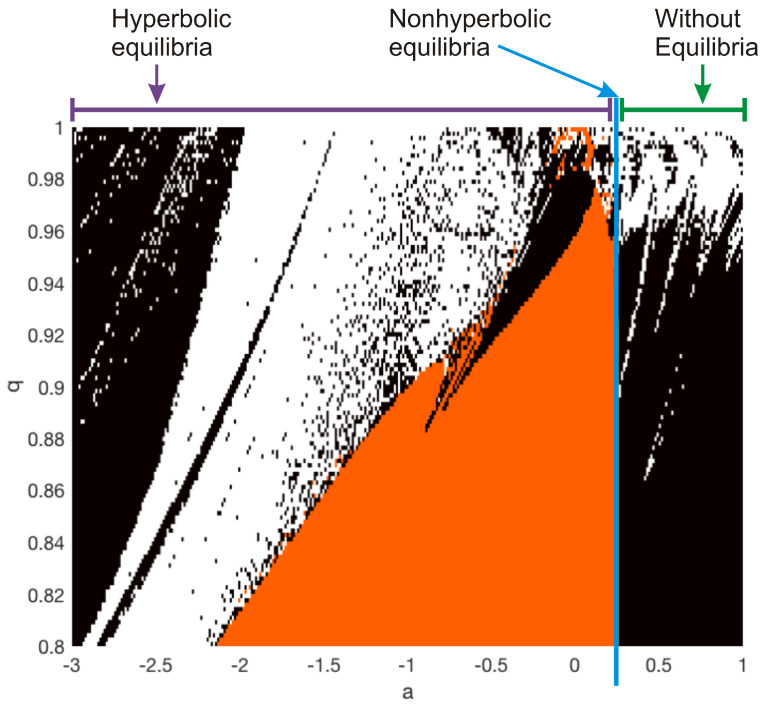
Bi-dimensional map for the different dynamical behaviors of the fractional-order system (15) as a function of the parameter *a* and order *q*. The white region leads to a chaotic attractor, the black region evolves to periodic attractors, and the orange region converges to unbounded orbits. Self-excited, nonhyperbolic, and hidden chaotic attractors for a<1/4, a=1/4, and a>1/4, respectively.

Finally, the fractional-order system (15) has no-equilibrium points when a>1/4. In this scenario, the resulting system can be categorized into a class of fractional-order no-equilibrium chaotic systems. It is interesting that if there are no-equilibrium points, the system (15) also presents multistability, since two distinct attractors are observed for different initial conditions. It is straightforward to observe that we added the simple constant control parameter *a* to the fractional-order chaotic system (15), trying to change the stability of its equilibria while preserving its chaotic dynamics. With the aim to analyze the relationship between the parameter *a* and the fractional-order *q*, we introduce the bi-dimensional map, that it is essentially a bifurcation diagram of two parameters, shown in Figure 9.

This map indicates the type of equilibrium and the resulting dynamical behavior for a given *a* and *q*. The white, black, and orange regions evolve in chaotic, periodic, and unbounded behavior, respectively. From Figure 9, the minimal fractional order can be also determined. For instance, when a=−1, we observe that the chaotic attractor can appear for q>0.9010, as was demonstrated in Section 3.1. However, unbounded trajectories are obtained if q≤0.9010, but a chaotic behavior can be detected for fractional orders as low as q=0.8 when a=−2.5. Similarly, the chaotic attractor from nonhyperbolic equilibria is found for q>0.997. For the case of a no-equilibria system (a>1/4), we observed that the minimal fractional order wherein hidden chaotic attractors can emerge is about q=0.955. For lower orders, the system only generates hidden periodic attractors.

To the best knowledge of the authors, this is the first time reporting a fractional-order chaotic system without equilibrium points and with the coexistence of hidden attractors.

## 4. Test 0–1 for Chaos

Gottwald and Melbourne [67] proposed a reliable and effective binary test method for testing whether a nonlinear system has chaotic behavior, which is called the “0–1 test”. The test consists of creating a random dynamic process for the data and then studying how the scale of the stochastic process changes with time [67,68,69]. This test has been widely adopted as a suitable tool to confirm the chaotic behavior in fractional-order dynamical systems [26,33,40] because it is binary (minimizing issues of distinguishing small positive numbers from zero); the nature of the vector field, as well as its dimensionality, does not pose practical limitations; and it does not suffer from the difficulties associated with phase space reconstruction.

In this manner, the “0–1 test” is applied directly to the time series data of the fractional-order system (15). Since the test does not require phase space reconstruction, the dimension and origin of the system (15) are irrelevant. Let us consider a set of discrete data ϕ(n) with n=1,2,…,N, representing a one-dimensional observable dataset obtained from the underlying dynamics of the system (15). For c∈(0,π), we compute the translation variables $p_{1}\left(n\right)=\sum_{j=1}^{n}\phi \left(j\right)\cos\left(jc\right)$, and $\;p_{2}\left(n\right)=\sum_{j=1}^{n}\phi \left(j\right)\sin\left(jc\right).$ Next, the diffusive or non-diffusive behavior of $p_{1}$ and $p_{2}$ is obtained by the mean square displacement $M\left(n\right)=\lim_{N\to \infty }\frac{1}{N}\sum_{j=1}^{N}\left({\left[p_{1}\left(j+n\right)-p_{1}\left(j\right)\right]}^{2}+{\left[p_{2}\left(j+n\right)-p_{2}\left(j\right)\right]}^{2}\right)$, for n≪N. Finally, the asymptotic growth rate *K* of M(n) is given by

(21) $$K=\lim_{n\to \infty }\frac{\log M(n)}{\log n}.$$

When M(n) is bounded, the dynamics of the system (15) evolves in a periodic or quasi-periodic behavior. On the other hand, a chaotic behavior is detected if M(n) grows linearly, similar to a Brownian motion. Moreover, a quantitative measure of the dynamics of the system (15) is given by *K*. For *K* close to 1, a chaotic behavior is observed, whereas for *K* close to 0, a regular behavior is obtained.

### Detecting Chaos in the Proposed Fractional-Order System

In order to determine the chaotic and regular behaviors in the fractional-order system (15), we apply the “0–1 test” to the time series data obtained from the different scenarios in Section 3. The time series data were obtained by the ABM scheme with a time-step size h=0.01.

**Figure 10:**
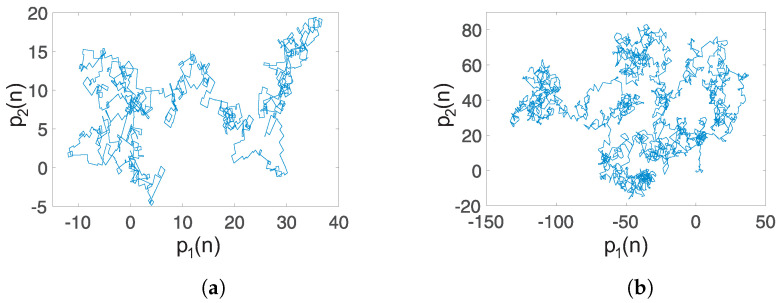
Dynamics of the translation components $\left(p_{1},p_{2}\right)$ of the fractional-order system (15): (**a**) Self-excited chaotic attractor (q=0.93, a=−1) with an asymptotic growth rate K=0.9988; (**b**) hidden chaotic attractor (q=0.97, a=0.35), with K=0.9985.

Case 1: Self-excited attractor: When q=0.93 and a=−1, the translation components $\left(p_{1},q_{1}\right)$ are as shown in Figure 10a. The unbounded behavior points out that the dynamics of the system (15) with unstable equilibria is chaotic. Also, the asymptotic growth rate *K* approaches one, with a value K=0.9988, indicating the presence of chaotic dynamics. This result agrees with the self-excited chaotic attractor shown in Figure 1.

Case 2: Hidden chaotic attractor: When q=0.97 and a=0.35, a hidden chaotic attractor is localized, as shown by the phase portraits in Figure 4. In this case, the asymptotic growth rate of the time series of the system (15) with no-equilibrium is K=0.9985. Additionally, the translation components $\left(p_{1},p_{2}\right)$ are shown in Figure 10b. The Brownian-like motion indicates chaotic behavior.

**Figure 11:**
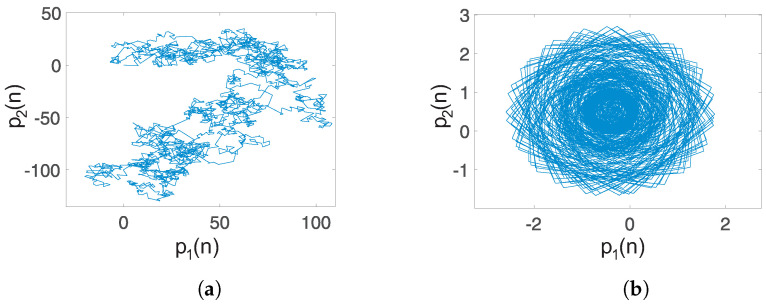
Dynamics of the translation components $\left(p_{1},p_{2}\right)$ of the fractional-order system (15): (**a**) Coexisting hidden chaotic attractor (q=0.996, a=0.35, (x,y,z)=(1,1,1)) with an asymptotic growth rate K=0.9975; (**b**) coexisting hidden periodic attractor (q=0.996, a=0.35, (x,y,z)=(0,75,−50) with K=0.0364.

Case 3: Coexistence of hidden attractors: When q=0.996, a=0.35, and initial conditions ${\left[1,1,1\right]}^{T}$, we localize a hidden chaotic attractor, as shown in Figure 7. By applying the “0–1 test”, K=0.9975. Besides, the translation components $\left(p_{1},p_{2}\right)$, shown in Figure 11a, behave as Brownian-like motion. When the initial conditions are chosen as ${\left[0,75,-50\right]}^{T}$, and the parameters a,q maintain the same value, the translation components $\left(p_{1},p_{2}\right)$ are now bounded, as shown in Figure 11b. Besides, the asymptotic growth rate is K=0.0364. Therefore, the hidden attractor is periodic.

From Case 1 to Case 3, the “0–1 test” proved that three different dynamics can arise in the fractional-order system (15), i.e., a self-excited chaotic attractor, a hidden chaotic attractor, and the coexistence of hidden attractors.

## 5. Spectral Entropy Analysis

Complexity measures are an important way to characterize the complex behavior of a chaotic system. In information security, the complexity can reflect the security of a system [32]. Currently, there are several methods to measure the complexity of a time series [70]. In this sense, the complexity of chaotic sequences can be divided into behavior complexity and structural complexity. The former measures the size of the probability of a new pattern for a short-time window, while the latter is used to measure the complexity of a sequence by its frequency characteristic and energy spectrum in the transformation domain. Compared with the behavior complexity, the structural complexity has a global statistical significance, because it focuses on analyzing the energy characteristic based on all but the local sequence [70]. At present, the algorithms to evaluate structural complexity include spectral entropy (SE) and $C_{0}$ entropy.

Herein, we choose the spectral entropy algorithm to calculate the corresponding Shannon entropy value based on the Fourier transformation of the time series of the fractional-order system (15). By removing the direct-current, the steps are as follows. Given the time series $\left\{x^{N}\left(n\right),n=0,1,2,\ldots ,N-1\right\}$ of the system (15) with length *N*, let $x\left(n\right)=x\left(n\right)-\bar{x},$, where $\bar{x}$ is the mean value of the time series, $\bar{x}=\frac{1}{N}\sum_{n=0}^{N-1}x\left(n\right)$. After that, the discrete Fourier transform (DFT) for the sequence x(n) is computed with $X\left(k\right)=\sum_{n=0}^{N-1}x\left(n\right)e^{-j2\pi nk/N}=\sum_{n=0}^{N-1}x\left(n\right)W_{N}^{nk}$, where k=0,1,2,…,N−1. Next, the relative power spectrum is derived with $P_{k}=\frac{{\left|X\left(k\right)\right|}^{2}}{\sum_{k=0}^{N/2-1}{\left|X\left(k\right)\right|}^{2}}$. By using x(n), X(k), and $P_{k}$, the spectral entropy of the time series of the system (15) for the scenarios in Section 3 can be determined by

(22) $$SE=\frac{\sum_{k=0}^{N/2-1}\left|P_{k}\ln P_{k}\right|}{\ln(N/2)},$$

where ln(N/2) is the entropy of a completely random signal.

### 5.1. Structural Complexity of the New Fractional-Order Chaotic System

**Figure 12:**
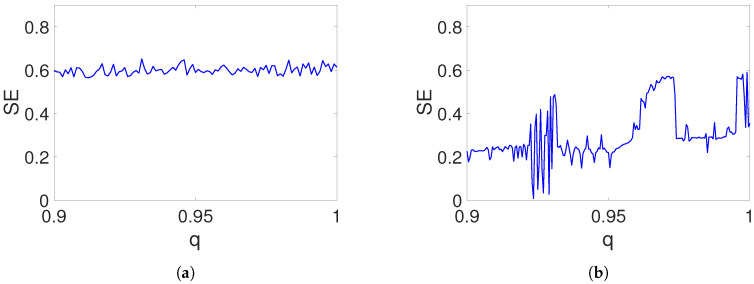
Spectral entropy versus fractional-order *q* for the system (15): (**a**) Structural complexity of the self-excited attractor in Figure 1 (a=−1); (**b**) structural complexity of the hidden chaotic attractor in Figure 4 (a=0.35).

The structural complexity of the self-excited and hidden attractors generated by the fractional-order system (15) is analyzed by Equation (22). The SE is computed from the time series x(n) of the system (15) with length $N=4.5\times 10^{4}$. Figure 12a shows the SE for the case of the self-excited attractor, whereas Figure 12b displays the SE of the hidden attractor. The complexity of the self-excited attractor is almost constant in the interval q∈[0.9,1]. On the other hand, the SE of the hidden attractor, as a function of fractional order, presents regions where the complexity is close to SE=0.6, but other regions have a low SE. Therefore, we must be aware of the selected fractional order in the hidden attractor in order to have a relatively high structural complexity.

### 5.2. Design of a PRNG Using Hidden Attractors

By considering the results of the structural complexity, we select the hidden attractor of the system (15) to design a pseudo-random number generator (PRNG). More specifically, the chaotic signals obtained from the system (15) with a=0.35 and q=0.97 are used to generate a bitstream using the approach in [33,71]. In this manner, the chaotic signal x(t) of the system (15) is sampled randomly to get samples $\gamma_{i}$ with a suitable sample space. A ceil function is required to convert the real value into an integer value. Next, from each sampled value, we obtain $g_{e}\left(o\right)$ of 4-bit resolution composed of the four least-significant bits. As a post-processing operation, the output bits g(o) are obtained XORing two consecutive $g_{e}\left(o\right)$.

The performance of the PRNG designed with the hidden dynamics is characterized by using the NIST SP 800-22 battery of statistical tests [72]. By selecting a confidence level α=0.01, the *p*-values are determined for sequences of 1 Mbit. As is well known, a *p*-value ≥0.01 means that the sequence is considered to be random with a confidence of 99%. Table 2 summarizes the results. As can be seen, the resulting PRNG using the hidden attractor of the system (15) satisfies all statistical tests.

## 6. Conclusions

In this paper, a fractional-order dynamical system with different families of hidden and self-excited attractors is introduced. As a function of only one parameter, the fractional-order system can be defined without equilibrium points, with nonhyperbolic equilibria, and with hyperbolic equilibria. A hidden chaotic attractor was identified in the proposed fractional-order system when it has no-equilibrium points. Additionally, it was found that two different attractors coexist for a determined fractional order, indicating multistability. Not only hidden dynamics were generated by the new system, but also two distinct self-excited chaotic attractors were obtained. Lyapunov exponents and the Brownian-like motion approach demonstrated the chaotic behavior of the system for each scenario. Finally, the structural complexity of the hidden and self-excited dynamics were evaluated using the spectral entropy. As an application, a PRNG with a suitable performance was designed with the time series of the hidden chaotic attractor.

As consequence, a contribution to this new phenomenon and little-explored area is the description of a fractional-order chaotic no-equilibrium system, along with the coexistence of hidden attractors. Such nonlinear systems without equilibrium and with multistability are appropriate for practical applications. Moreover, the fractional order is an extra parameter that permits the study of dynamical behaviors with more accuracy.

## Figures and Tables

**Table 1:** Equilibria, eigenvalues, and Lyapunov exponents of the fractional-order chaotic system (15).

New System	Parameters	FO	Equilibria	Eigenvalues	$x_{0},y_{0},z_{0}$	LEs
Self-excited	a=−1;	q=0.93	(1,1.6180, −1.6180)	2.3064,−0.3442±0.9225i	(1,1,1)	$LE_{1}=2.957$
			(−1,−0.6180,−0.6180)	−1.0666,0.2243±1.4304i		$LE_{2}=0.01$
			(1,−0.6180,0.6180)	−1.0666,0.2243±1.4304i		$LE_{3}=-5.765$
			(−1,1.6180,1.6180)	2.3064,−0.3442±0.9225i		
Non-hyperbolic	a=0.25;	q=0.99	$\left(1,\frac{1}{2},-\frac{1}{2}\right)$	0,−0.3750+0.5994i	(1,1,1)	$LE_{1}=1.27$
			$\left(-1,\frac{1}{2},\frac{1}{2}\right)$	0,−0.3750+0.5994i		$LE_{2}=0.010$
						$LE_{3}=-1.72$
Hidden	a=0.35;	q=0.97	no-equilibria		(1,1,1)	$LE_{1}=14.735$
						$LE_{2}=0.010$
						$LE_{3}=-18.350$
Coexistence Chaotic	a=0.35;	q=0.996	no-equilibria		(1,1,1)	$LE_{1}=11.066$
						$LE_{2}=0.080$
						$LE_{3}=-13.161$
Coexistence Periodic	a=0.35;	q=0.996	no-equilibria		(0,75,−50)	$LE_{1}=0$
						$LE_{2}=-3.695$
						$LE_{3}=-3.705$

**Table 2:** Results of NIST statistical tests for the bit sequences based on the system (15) when it presents a hidden chaotic attractor.

Statistical Test	*p*-Value	Results
Frequency	0.654721	success
Block Frequency	0.420199	success
Cusum-Forward	0.600222	success
Cusum-Reverse	0.446686	success
Runs	0.220773	success
Long Runs of Ones	0.012522	success
Rank	0.254592	success
Spectral DFT	0.538167	success
Non-Overlapping Templates	0.615839	success
Overlapping Templates	0.102065	success
Universal	0.830304	success
Approximate Entropy	0.635119	success
Random Excursions	0.407574	success
Random Excursions Variant	0.444982	success
Linear Complexity	0.634990	success
Serial	0.301388	success
